# The Relationship Between Metabolic Risk Factors and Incident Cardiovascular Disease in Europeans, South Asians, and African Caribbeans: SABRE (Southall and Brent Revisited)—A Prospective Population-Based Study

**DOI:** 10.1016/j.jacc.2012.12.046

**Published:** 2013-04-30

**Authors:** Therese Tillin, Alun D. Hughes, Jamil Mayet, Peter Whincup, Naveed Sattar, Nita G. Forouhi, Paul M. McKeigue, Nish Chaturvedi

**Affiliations:** ⁎ International Centre for Circulatory Health, National Heart and Lung Institute, Imperial College London, London, United Kingdom; † Division of Population Health Sciences and Education, St. George's University of London, London, United Kingdom; ‡ Metabolic Medicine, University of Glasgow School of Medicine, Glasgow, United Kingdom; § MRC (Medical Research Council) Epidemiology Unit, Institute of Metabolic Science, University of Cambridge, Cambridge, United Kingdom; ∥ Centre for Population Health Sciences, University of Edinburgh, Edinburgh, United Kingdom

**Keywords:** coronary heart disease, ethnicity, incidence, stroke, CHD, coronary heart disease, CVD, cardiovascular disease, HDL, high-density lipoprotein, ICD, International Classification of Diseases, IR, insulin resistance, LDL, low-density lipoprotein, SHR, subhazard ratio

## Abstract

**Objectives:**

This study sought to determine whether ethnic differences in diabetes, dyslipidemia, and ectopic fat deposition account for ethnic differences in incident cardiovascular disease.

**Background:**

Coronary heart disease risks are elevated in South Asians and are lower in African Caribbeans compared with Europeans. These ethnic differences map to lipid patterns and ectopic fat deposition.

**Methods:**

Cardiovascular risk factors were assessed in 2,049 Europeans, 1,517 South Asians, and 630 African Caribbeans from 1988 through 1991 (mean age: 52.4 ± 6.9 years). Fatal and nonfatal events were captured over a median 20.5-year follow-up. Subhazard ratios (SHR) were calculated using competing risks regression.

**Results:**

Baseline diabetes prevalence was more than 3 times greater in South Asians and African Caribbeans than in Europeans. South Asians were more and African Caribbeans were less centrally obese and dyslipidemic than Europeans. Compared with Europeans, coronary heart disease incidence was greater in South Asians and less in African Caribbeans. The age- and sex-adjusted South Asian versus European SHR was 1.70 (95% confidence interval [CI]: 1.52 to 1.91, p < 0.001) and remained significant (1.45, 95% CI: 1.28 to 1.64, p < 0.001) when adjusted for waist-to-hip ratio. The African Caribbean versus European age- and sex-adjusted SHR of 0.64 (95% CI: 0.52 to 0.79, p < 0.001) remained significant when adjusted for high-density lipoprotein and low-density lipoprotein cholesterol (0.74, 95% CI: 0.60 to 0.92, p = 0.008). Compared with Europeans, South Asians and African Caribbeans experienced more strokes (age- and sex-adjusted SHR: 1.45 [95% CI: 1.17 to 1.80, p = 0.001] and 1.50 [95% CI: 1.13 to 2.00, p = 0.005], respectively), and this differential was more marked in those with diabetes (age-adjusted SHR: 1.97 [95% CI: 1.16 to 3.35, p = 0.038 for interaction] and 2.21 [95% CI: 1.14 to 4.30, p = 0.019 for interaction]).

**Conclusions:**

Ethnic differences in measured metabolic risk factors did not explain differences in coronary heart disease incidence. The apparently greater association between diabetes and stroke risk in South Asians and African Caribbeans compared with Europeans merits further study.

Cardiovascular disease (CVD) is now the leading cause of death globally (1). Marked ethnic differences in CVD exist, highlighted by a comparison of migrant and host populations. Mortality resulting from coronary heart disease (CHD) and stroke in South Asian migrants to the United Kingdom are 50% to 100% higher than the general United Kingdom population (2), mirroring risks in the Indian subcontinent (3). In contrast, people of black African and African Caribbean origin enjoy significant protection from CHD in the United Kingdom, although stroke mortality rates are even higher than those of South Asians (2). These observations reflect historical risks in black African migrants to the United States (4) and in Africa itself. These ethnic differentials in mortality have not been explained (5,6). However, previous analyses limited to deaths may be misleading.

Both ethnic minority groups have more insulin resistance (IR) and diabetes than Europeans, but although South Asians display classical dyslipidemia and central obesity associated with IR, African Caribbeans have favorable lipoprotein profiles and less central obesity than Europeans. We hypothesized that diabetes and associated metabolic disturbances, measured in midlife, would account for ethnic differences in incident fatal and nonfatal CVD in a unique tri-ethnic community-based United Kingdom cohort followed for 20 years.

## Methods

**Figure 1:**
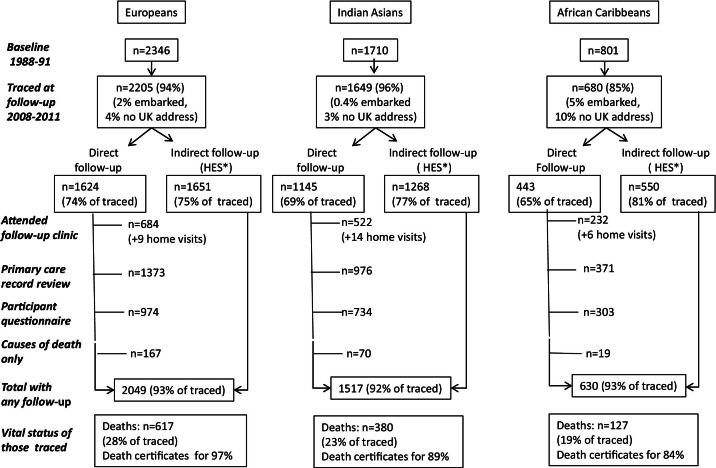
Follow-Up of SABRE Study Cohort (2008 Through 2011) Flowchart showing status and responses at the 20-year follow-up. SABRE = Southall and Brent Revisited.

The SABRE (Southall and Brent Revisited) study examined a tri-ethnic community-based cohort from North and West London. Details of the cohort have been published (7). Briefly, participants 40 to 69 years of age at baseline (1988 through 1991) were selected randomly from 5-year age- and sex-stratified primary care physician lists (n = 4,063) and workplaces (n = 795) in the London districts of Southall and Brent (Fig. 1). The baseline studies initially were designed to study ethnic differences in metabolic risk factors in association with CVD in men; however, as the studies progressed, the importance of CVD in women was recognized and later recruitment included women. Because African Caribbeans were recruited a little later into the study, the gender rebalance was more complete in this than in the other ethnic groups (7). Ethnicity was agreed on with the interviewer at baseline based on self-report, parental place of origin, and appearance. All South Asians and black African and African Caribbeans were migrants. Most African Caribbeans (92.5%) were born in the Caribbean, and the remainder were born in West Africa. We previously reported similar cardiometabolic risks in these latter 2 groups (8). Most (82%) South Asians were born in the Indian subcontinent, and 14% were born in East Africa. Just more than half (52%) were of Punjabi Sikh origin.

Participants attended a baseline clinic after an overnight fast. They underwent blood pressure measurements, electrocardiography, and anthropometry and completed a health and lifestyle questionnaire (7). Height was measured using a stadiometer. Body fat measurements included waist (halfway between costal margin and iliac crest), hip (over greater trochanter), and mid-thigh circumferences.

Fasting bloods were drawn, and those not known to have diabetes underwent an oral glucose tolerance test (7). Bloods were analyzed for glucose, insulin, and lipids at the same hospital laboratory (7). Glycated hemoglobin was measured on stored whole blood samples (Southall center only) using an immunoassay on a clinically validated automated analyzer (c311, Roche, Burgess Hill, United Kingdom). Apolipoproteins were measured on available stored aliquots in a subsample of 2,349 participants (1,147 [56%] Europeans, 688 [45%] South Asians, and 514 [82%] African Caribbeans) using immunoturbidimetric methods.

Baseline IR was estimated using the homeostasis model assessment 2 calculator (9). Baseline diabetes was defined according to World Health Organization criteria (10), self-report of doctor-diagnosed diabetes, or receipt of antidiabetes medications. Seated resting blood pressure was measured using a random zero sphygmomanometer (Hawksley, London, United Kingdom). Two measurements were obtained, and the mean of these was used in all analyses. Physical activity was summarized as the total weekly energy expended (in megajoules) in sports, walking, and cycling using questions and energy expenditure estimates based on the Allied Dunbar Fitness survey questionnaire (11) and earlier work by Durnin and Passmore (12,13). Frequency of fruit and green vegetable consumption was assessed by a simple dietary questionnaire. The Registrar General's Classification of Occupations (14) was used to assign midlife occupational status as manual or nonmanual.

Since baseline, participants have been flagged for death by the Office for National Statistics. From 2008 through 2011, survivors were invited to take part in a morbidity follow-up. This included a health and lifestyle questionnaire, primary care medical record review, attendance at our clinic at St. Mary's Hospital, London, or a combination thereof. Hospital Episode Statistics were obtained for traced participants since baseline.

All participants gave written informed consent. Approval for the study at baseline was obtained from Ealing, Hounslow, and Spelthorne, and University College London research ethics committees, and at follow-up from St. Mary's Hospital Research Ethics Committee (ref. 07/H0712/109).

### Identification of first post-baseline CHD and stroke events

For CHD, a composite endpoint comprised the first event after baseline identified from any of the following sources:

1 Cause of death included any of the following: angina, myocardial infarction or its sequelae, or atherosclerotic heart disease using International Classification of Disease-Ninth Edition (ICD-9) codes 410 through 415 or ICD-Tenth Edition (ICD-10) codes I200 through I259.
2 Primary care data were reviewed independently by 2 senior physicians blinded to participant ethnicity and identity. A CHD event was identified if both physicians agreed on definite or probable diagnosis of myocardial infarction or acute coronary syndrome, according to pre-determined criteria used in the ASCOT (Anglo-Scandinavian Cardiac Outcomes Trial) (15), based on symptoms, cardiac enzymes, electrocardiography findings, and hospital discharge diagnosis. Adjudication by a third physician was conducted if required. Coronary interventions (coronary artery bypass graft, angioplasty, stenting) were included as incident CHD events, as was angina confirmed on exercise testing.
3 Hospital Episode Statistics: for causes of death, diagnostic ICD-9 codes 410 through 415 or ICD-10 codes I200 through I259 were used, in addition to any of the following operation codes from the Office of Populations and Surveys classification of interventions and procedures: K401 through K469, K491 through K504, K751 through K759, or U541 (coronary revascularization interventions or rehabilitation for ischemic heart disease).

For stroke, the first event after baseline identified from any of the following sources:

1 Cause of death included ICD-9 codes 430 through 439 or ICD-10 codes I600 through I698.
2 Primary care data were reviewed in a similar manner to CHD, with definite or probable diagnosis of stroke made according to pre-determined criteria based on symptoms, duration of symptoms, and magnetic resonance image or computed tomography imaging.
3 Hospital Episode Statistics: diagnostic ICD-9 codes 430 through 439 or ICD-10 codes I600 through I698.
4 Participant report of physician-diagnosed stroke and duration of symptoms in excess of 24 h.

Coronary heart disease or stroke that occurred before baseline was identified from participant report (at baseline) of physician-diagnosed disease (or presence of major Q waves on baseline electrocardiography for CHD).

### Statistical analyses

Primary analyses related to participants with follow-up data from any source. We combined first post-baseline fatal and nonfatal CHD or stroke events as primary outcomes. Baseline characteristics were stratified by sex; ethnic group comparisons were made with Europeans as the reference group within each sex, using parametric (Student *t*) or nonparametric (Wilcoxon rank-sum or chi-square) statistical tests as appropriate.

Competing risk regression (competing risk = death from other causes) based on the proportional subhazards methods of Fine and Gray (16) was used to describe ethnic differences in incidence of primary outcomes. A priori testing revealed significant interactions between ethnicity and baseline diabetes for both South Asians and African Caribbeans compared with Europeans in stroke prediction. Hence, we show models predicting stroke events stratified by baseline diabetes status.

We assessed linearity of associations using tertiles of continuous covariates; no initial analyses suggested departure from linearity, and we included these as linear terms, with exceptions of blood pressure, where we used 4 categories (those receiving treatment in the top category and the remaining into thirds by ascending level of blood pressure). We grouped measures of glycemia and IR similarly, with diabetes in the top category. There were no significant sex and ethnicity interactions in association with stroke or CHD events, and we show results for men and women combined to maximize statistical power. We investigated potential mediators of observed ethnic differences in univariate analyses. Multivariable models included covariates that had the greatest positive or negative effects on ethnic differentials. We examined the composite Framingham (17) and INTERHEART (18) study risk factors for stroke and CHD outcomes.

To assess the integrity of proportional hazards assumptions, we tested interactions between covariates and follow-up time. We plotted cumulative incidence curves for each ethnic-diabetes group and examined Schoenfeld-like residuals.

### Sensitivity analyses

We compared baseline characteristics of 661 people lost to follow-up with those of 4,196 people followed-up. We repeated the above analyses as follows: (1) excluding 47 people who migrated directly from West Africa; (2) using data derived from direct follow-up only (i.e., without Hospital Episode Statistics data); (3) separately for fatal and nonfatal events; (4) excluding participants with baseline CHD or stroke; and (5) including diabetes identified during follow-up and before events in multivariable analyses and stratification. All analyses were conducted in Stata software version 12.0 (Stata Corporation, College Station, Texas). Statistical significance was accepted as p < 0.05.

## Results

We traced 4,534 participants (93%) to a United Kingdom address. Follow-up data were obtained for 4,196 participants (>92%) (Fig. 1). Participants were followed up for a median of 20.5 years.

### Baseline (1988 through 1991)

Follow-up data were available for 2,049 Europeans (24% women), 1,517 South Asians (17% women), and 630 African Caribbeans (45% women). At baseline, the mean age of participants was 52.4 years (standard deviation: 6.9 years, range: 40 to 70 years). Baseline stroke was infrequent in all ethnic groups, and African Caribbean men had less CHD than European men. As expected, South Asians and African Caribbeans had more diabetes and hypertension than Europeans. Although they had lower body mass indices than European men, South Asian men were more centrally obese and dyslipidemic. African Caribbeans were less dyslipidemic than Europeans and African Caribbean men were less centrally obese than European men. South Asians and African Caribbeans smoked less and consumed less alcohol than Europeans (Table 1).

There was more loss to follow-up in African Caribbeans (21%) than in Europeans (13%) or South Asians (11%). Baseline characteristics of participants lost to follow-up were similar within the ethnic group when compared with those who were followed up (Online Appendix, Online Table 1).

### CHD events during follow-up (to 2011)

**Figure 2:**
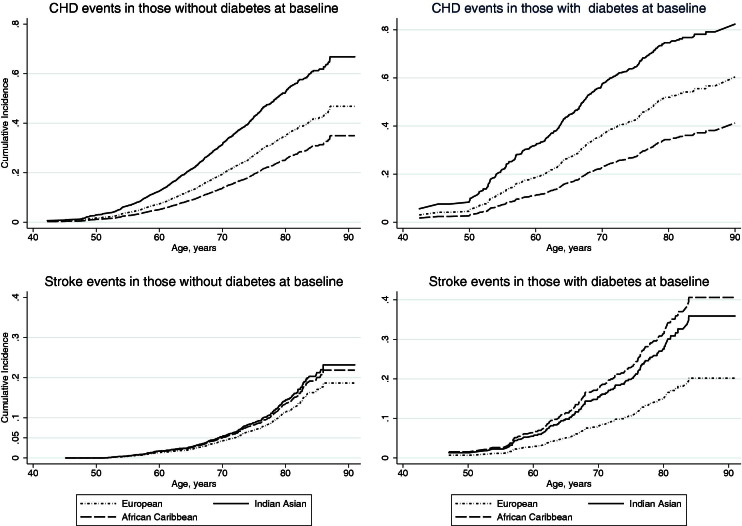
Cumulative Incidence of Coronary Heart Disease and Stroke Events Cumulative incidence curves for combined fatal and nonfatal events, adjusted for age, sex, smoking, and baseline coronary heart disease (CHD) and stroke.

Coronary heart disease events occurred in 1,256 (30%) participants. Fatal CHD was the first recorded follow-up event in 159 participants. South Asians were most and African Caribbeans least at risk for CHD (Fig. 2). Incidence rates increased with age in all ethnic groups (Table 2). South Asians were on average 2.3 years younger at first post-baseline CHD event than Europeans (63.9 ± 8.6 years vs. 66.2 ± 8.6 years, p < 0.001), whereas African Caribbeans were 2.4 years older (68.6 ± 7.5 years, p = 0.008). Angina (exercise test confirmed) comprised 3.5% of first CHD events, whereas coronary revascularization procedures comprised the first event in 43 (7.8%) Europeans, 51 (8.5%) South Asians, and 2 (1.9%) African Caribbeans; the remainder of first events were identified as acute ischemic events or were related to atherosclerotic heart disease.

The age- and sex-adjusted subhazard ratio (SHR) for CHD between South Asians and Europeans was 1.70 (95% confidence interval [CI]: 1.52 to 1.91, p < 0.001) (Table 3). Of all measured individual risk factors, waist-to-hip ratio best attenuated the South Asian excess risk, although risk remained significantly elevated compared with that of Europeans (SHR: 1.45, 95% CI: 1.28 to 1.64, p < 0.001). Other attenuating factors included diabetes and measures of glycemia, IR, triglyceride, and HDL cholesterol. Adjustment for both protective (lower smoking prevalence) and adverse (waist-to-hip ratio and glycosylated hemoglobin) factors (adjusted SHR: 1.49, 95% CI: 1.25 to 1.77, p < 0.001) did not account for the South Asian CHD excess, nor did adjustment for Framingham or INTERHEART factors (Table 3). Markers of baseline glycemic status were (nonsignificantly) more predictive of CHD events in South Asians than in Europeans.

CHD risk was lower in African Caribbeans than Europeans (age- and sex-adjusted SHR: 0.64, 95% CI: 0.52 to 0.79, p < 0.001). Our multivariate model, including favorable and unfavorable risk factors (HDL and LDL cholesterol, waist-to-thigh ratio, blood pressure, age, sex), did not account for the African Caribbean protection from CHD (adjusted SHR: 0.74, 95% CI: 0.59 to 0.93, p = 0.01) (Table 3). Baseline diabetes was associated most strongly with CHD events in South Asians and was associated least strongly in African Caribbeans. The age- and sex-adjusted SHR for diabetes were as follows: South Asians: 1.90 (95% CI: 1.59 to 2.27), Europeans: 1.61 (95% CI: 1.23 to 2.10), and African Caribbeans: 1.31 (95% CI: 0.85 to 2.02).

Findings from sensitivity analyses were similar to those presented here.

### Stroke events during follow-up (to 2011)

Stroke events occurred in 401 participants during follow-up. African Caribbeans had the highest and Europeans the lowest rates of stroke (Table 2).

In South Asians and African Caribbeans, the age- and sex-adjusted SHR, 1.45 (95% CI: 1.17 to 1.80, p = 0.001) and 1.50 (95% CI: 1.13 to 2.00, p = 0.005), respectively, were strongly but incompletely attenuated on adjustment for baseline diabetes (SHR: 1.27 [95% CI: 1.02 to 1.58, p = 0.03] and 1.33 [95% CI: 1.01 to 1.76, p = 0.044]).

Diabetes was predictive of stroke in all ethnic groups (Fig. 2), but most profoundly in African Caribbeans, in whom diabetes was associated with a 3.0-fold (95% CI: 1.8 to 4.8) age-adjusted incidence of stroke compared with a 1.3-fold (95% CI: 0.8 to 2.1) age-adjusted incidence of stroke in Europeans (p = 0.019 for ethnicity and diabetes interaction) (Online Appendix, Online Table 2). A similar interaction was observed for South Asians, in whom diabetes was associated with a 2.5-fold (95% CI: 1.8 to 3.4) incidence of stroke (p = 0.038 for interaction) (Online Table 2).

In people with diabetes, the age-adjusted SHR in South Asians versus Europeans, at 1.96 (95% CI: 1.15 to 3.33, p = 0.013), was little affected on adjustment for any measured risk factors. Multivariable adjustment (smoking, years of education) had a small attenuating effect, whereas Framingham and INTERHEART adjustments did not explain the South Asian excess in those with diabetes. In African Caribbeans with diabetes, the age-adjusted SHR (2.30, 95% CI: 1.25 to 4.22, p = 0.007) was little changed on multivariable adjustment. The most marked attenuation was obtained by the INTERHEART adjustment (adjusted SHR: 1.62, 95% CI: 0.71 to 3.65, p = 0.25). In those without diabetes, there was only a modest and nonsignificant ethnic excess in risk of stroke (Table 4).

The diabetes and ethnicity interactions also were strong on analyses of fatal stroke events only. Date of diagnosis of pre-baseline diabetes was available for 440 (76%) participants with baseline diabetes; however, adjustment for age at diagnosis in these participants did not alter the ethnic differentials in stroke events. Sensitivity analyses demonstrated similar magnitude and direction of ethnicity-associated excess stroke risk and similar diabetes and ethnicity interactions. Inclusion of new diabetes (identified during follow-up and before stroke) in addition to baseline diabetes in multivariable analyses confirmed the additional risk of stroke in South Asians and African Caribbeans with diabetes compared with Europeans with diabetes, with age- and sex-adjusted SHR as follows: South Asians: 2.10 (95% CI: 1.34 to 3.30, p = 0.001), and African Caribbeans: 2.17 (95% CI: 1.28 to 3.68, p = 0.004).

## Discussion

People of European, South Asian, and African Caribbean origin vary markedly in CVD risk, in parallel with differences in metabolic factors such as IR, dyslipidemia, and central adiposity. However, the between-ethnic group differences in CVD remained even after adjustment for conventional cardiometabolic risk factors measured in midlife. It is well recognized that explanations for disease risk within a group may not explain differences between groups (19).

In 20 years of follow-up, CHD rates were significantly elevated in South Asian migrants compared with British Europeans. Multivariable adjustment for baseline risk factors explained little of the excess risk. In African Caribbeans (also migrants) rates of CHD continue to be 35% lower than in comparable Europeans, with adjustment for favorable midlife risk factors, such as HDL and LDL cholesterol, accounting for little of this protection. Rates of stroke remain elevated in both ethnic minority groups and more markedly so in people with diabetes in midlife, being 2.0 times greater in African Caribbeans and 1.7 times greater in South Asians compared with Europeans with diabetes.

There are few population-based studies comparing cardiovascular risk in South Asians and Europeans (20). To our knowledge, SABRE is the only one to have published on longitudinal associations between risk factors and CHD events. We extend our earlier reports, confined to fatal events (5), showing that greater case fatality in South Asians is unlikely to be the explanation for their excess CHD. Metabolic risk factors accounted for approximately one third of the excess CHD risk in South Asians. Residual confounding, specifically imprecise measurement of key risk factors, could explain why more of the South Asian excess could not be explained by metabolic risk factors. Single measurements of risk factors in midlife would be poorly representative of lifetime exposure (21). Additionally, factors acting at specific critical periods of the life course, for example in utero and infancy, may play a strong and independent role in determining adult risk (22). The INTERHEART study showed that just 9 risk factors account for most of the population-attributable risk of CHD, even in South Asian populations (23), and of these, the main roles were played by lipids and smoking. Adjustment for these factors did not account for interethnic differences in our study. However, although we have fasting HDL and LDL cholesterol readings for our entire study population, we have data for apolipoproteins only on a subsample. Nevertheless, risk estimates on multivariable adjustment for the apolipoprotein subset were identical to those yielded by multivariable analysis on the full dataset using the HDL-to-LDL ratio as a proxy for lipoproteins, confirming that known clusters of risk factors identified from other studies do not account for the ethnic difference in CHD. We do not have comparable measures of psychosocial stress at baseline, but the likelihood that the association between stress and myocardial infarction in the INTERHEART study is the result of reverse causality (i.e., the infarct increasing stress levels rather than the reverse) cannot be discounted. It is tempting to suggest a role for genetic factors, and although these cannot be discounted, it is notable that, to date, no such factor has been identified (24). Further, CHD rates have escalated in India in the last 50 years, with marked urban and rural differences, suggesting that, as in the West, CHD is a consequence of industrialization and is associated with reduced physical activity and an adverse diet (3). Genetic factors alone are unlikely to have acted this rapidly. South Asian migrants to the United Kingdom will have grown up in circumstances where infant mortality was high, malnutrition rife, and infectious disease endemic (25). Migration to an obesogenic environment in the 1950s and 1960s, the like of which is only now emerging in India today, will have compounded those early insults. Epigenetic alterations in response to exposures can determine phenotype, perhaps dependent on genotype, offering an attractive explanation for the marked increase in cardiometabolic disease in South Asians (26,27). Similarly, we show that the African Caribbean protection from CHD is not wholly explained by conventional risk factors measured in middle age, although the greatest attenuation is observed on adjustment for the highly favorable lipid patterns of African Caribbeans, despite an excess of diabetes and IR. Again, residual confounding resulting from lack of longitudinal measures and early life influences on development and growth may play a role. Protection from CHD also was observed in African Americans in the early part of the last century (4), although this protection has been somewhat eroded (28), implicating the impact of changes in environmental risk factors.

Our previous analyses hinted at a potential excess stroke mortality associated with diabetes in African Caribbeans (6). We have now taken these earlier analyses forward with longer follow-up and the inclusion of nonfatal events that continue to suggest that diabetes and dysglycemia may have more potent roles as precursors of stroke in both ethnic minorities. This association has not been reported, or perhaps sought previously, and was not observed for CHD, so it must be treated with caution until it is confirmed or refuted in other datasets. If confirmed, this would identify potential diabetes-related mechanisms in understanding ethnic differences in stroke. A potential candidate is the impaired cerebral autoregulation in both African Caribbeans and South Asians that we have noted as a consequence of autonomic dysfunction resulting from hyperglycemia and IR (29,30). This could contribute to excess stroke in minority ethnic groups. Duration of exposure to hyperglycemia also may contribute, although we did not demonstrate a significant explanatory effect of age at diagnosis in this study population. Measurement of age at diagnosis of diabetes is imprecise, so we cannot discount duration of exposure to hyperglycemia as an explanation for the greater toxicity of diabetes for stroke risk in ethnic minority groups.

### Study limitations

To our knowledge, this is the largest tri-ethnic cohort in the United Kingdom with a 20-year follow-up between middle and older age, thus providing valuable and unique prospective ethnicity-specific information on CHD and stroke incidence. Our combined data sources for the primary analyses provide follow-up data on more than 90% of the original study population, and although loss to follow-up was more frequent in African Caribbeans (21%), baseline characteristics of those lost to follow-up were similar to those participants who were followed up. Although this is a relatively large cohort, the numbers of stroke events were small, in particular and with the exception of incident diabetes, our baseline measurements were limited to those made on only 1 occasion 20 years ago, meaning that we cannot account for changes in other risk factors during the follow-up period or in earlier life. We were unable to differentiate reliably between stroke subtypes, which may vary by ethnicity, although it is reported that the risks of all stroke types are elevated in South Asian and African Caribbean populations and that ischemic strokes are most frequent in all 3 groups (31–35). Classification of causes of death and hospital discharge codes may be imprecise, but sensitivity analyses of fatal events alone and nonfatal events restricted to those ascertained from primary care record review or participant report result in remarkably similar findings as those reported here. We also should point out that more than half of South Asians in our study population were of Punjabi Sikh origin, and although most South Asian populations are at increased risk of diabetes and CVD, our findings may not apply to all South Asians. Finally, South Asians and African Caribbeans who migrated to the United Kingdom in the second half of the 20th century largely did so for economic reasons, and they may not be wholly representative of their countries of origin or of migrants to other countries.

## Conclusions

Morbidity and mortality resulting from CHD are elevated in South Asians and are lower in African Caribbeans compared with European-origin populations. These differences were not explained by conventional risk factors measured in midlife. Factors across the life course, in particular the mismatch between early and later life environments in migrant cohorts, may be key (36). This is of critical importance in lower income countries, where CHD risks are increasing, and in African Caribbean populations, where there is evidence that protection from CHD erodes with time in industrialized environments. Diabetes may be associated more strongly with stroke risk in both minority ethnic groups, and although further confirmation is needed in larger studies, we suggest that early interventions to reduce cardiovascular risk may be of particular importance in these high-risk populations.

## Figures and Tables

**Table 1:** Baseline Characteristics (1988 Through 1991), Unadjusted

	Men			Women		
	European	South Asian	African Caribbean	European	South Asian	African Caribbean
n	1,564	1,259	347	485	258	283
Age (yrs)	53.0 ± 7.1	51.1 ± 7.0, p < 0.001	53.6 ± 5.8, p = 0.141	53.2 ± 6.8	50.3 ± 6.5, p < 0.001	52.8 ± 6.1, p = 0.48
Diabetes	112 (7)	278 (22), p < 0.001	64 (18), p < 0.001	21 (4)	44 (17), p < 0.001	60 (21), p < 0.001
Treated hypertension	139 (9)	169 (13), p < 0.001	69 (20), p < 0.001	55 (11)	34 (13), p = 0.46	79 (28), p < 0.001
Known CHD	115 (8)	86 (7), p = 0.59	9 (3), p = 0.001	17 (4)	3 (1), p = 0.060	10 (4), p = 0.98
Known stroke	21 (1)	22 (2), p = 0.38	11 (3), p = 0.018	8 (2)	5 (2), p = 0.76	7 (2), p = 0.43
SBP (mm Hg)	123 ± 17	125 ± 17, p < 0.001	128 ± 17, p < 0.001	120 ± 17	125 ± 21, p < 0.001	131 ± 17, p < 0.001
DBP (mm Hg)	77 ± 11	81 ± 10, p < 0.001	81 ± 12, p < 0.001	75 ± 10	77 ± 10, p = 0.010	82 ± 12, p < 0.001
Waist circumference (cm)	92.1 ± 11.1	93.3 ± 9.6, p = 0.002	89.7 ± 10.2, p < 0.001	80.2 ± 12.2	86.2 ± 10.7, p < 0.001	88.9 ± 11.6, p < 0.001
Waist-to-hip ratio	0.94 (0.90–0.99)	0.98 (0.94–1.02), p < 0.001	0.94 (0.90–0.99), p = 0.89	0.79 (0.75–0.85)	0.87 (0.81–0.93), p < 0.001	0.86 (0.81–0.92), p < 0.001
Body mass index (kg/m^2^)	26.2 ± 3.9	25.9 ± 3.3, p = 0.008	26.4 ± 3.3, p = 0.44	26.1 ± 4.6	27.5 ± 4.6, p < 0.001	29.4 ± 5.0, p < 0.001
Weight (kg)	78.5 (71.0, 87.0)	73.8 (67.1–81.4), p < 0.001	77.5 (69.2–85.1), p = 0.067	65.5 (58.5–73.9)	64.6 (57.4–72.4), p = 0.100	74.6 (67.0–84.0), p < 0.001
Height (cm)	174.3 ± 6.9	169.8 ± 6.6, p < 0.001	171.9 ± 6.9, p < 0.001	161.2 ± 6.4	154.7 ± 5.6, p < 0.001	161.2 ± 5.5, p = 0.86
Total cholesterol (mmol/l)	6.0 (5.3–6.8)	5.9 (5.2–6.6), p = 0.002	5.4 (4.8–6.3), p < 0.001	6.0 (5.2–6.9)	5.7 (5.0–6.5), p < 0.001	5.6 (4.8–6.5), p < 0.001
Triglycerides (mmol/l)	1.5 (1.0–2.1)	1.8 (1.2–2.6), p < 0.001	1.1 (0.8–1.5), p < 0.001	1.3 (0.9–1.8)	1.4 (1.1–1.9), p < 0.001	1.1 (0.8–1.4), p < 0.001
HDL cholesterol (mmol/l)	1.2 (1.1–1.5)	1.1 (1.0–1.3), p < 0.001	1.4 (1.2–1.7), p < 0.001	1.6 (1.3–1.9)	1.4 (1.2–1.6), p < 0.001	1.6 (1.4–1.9), p = 0.61
Apolipoprotein B-to-A1 ratio	0.69 (0.56–0.83), n = 6,700	0.73 (0.61–0.85), n = 451	0.56 (0.44–0.71), n = 240	0.55 (0.45–0.67), n = 477	0.60 (0.49–0.70), n = 237	0.51 (0.39–0.64), n = 274
Fasting glucose (mmol/l)	5.4 (5.1–5.9)	5.6 (5.2–6.4), p < 0.001	5.6 (5.2–6.4), p < 0.001	5.3 (4.9–5.7)	5.1 (4.7–5.5), p = 0.003	5.6 (5.1–6.3), p < 0.001
Fasting insulin (μIU/ml)	7.5 (5.0–10.9)	10.8 (7.3–15.7), p < 0.001	8.7 (5.7–12.4), p < 0.001	5.3 (3.8–8.1)	7.7 (5.3–11.5), p < 0.001	9.3 (6.2–13.0), p < 0.001
HbA1c (%)	5.6 (5.4–5.8), n = 1,233	5.9 (5.6–6.3), p < 0.001, n = 939		5.5 (5.3–5.8), n = 189	5.8 (5.5–6.1), p < 0.001, n = 212	—
HOMA2 IR	0.9 (0.6–1.3)	1.2 (0.8–1.9), p < 0.001	1.0 (0.7–1.5), p < 0.001	0.6 (0.4–1.0)	0.9 (0.6–1.3), p < 0.001	1.1 (0.7–1.5), p < 0.001
Physical activity (leisure time; MJ/week)	4.0 (1.5–6.1)	3.5 (1.0–4.0), p < 0.001	3.7 (1.2–4.5), p < 0.001	3.7 (1.2–4.4)	1 (1–3.5), p = 0.001	3.7 (1.2–4.1), p = 0.46
Smoking categories, current/former/never (%)	34/40/26	16/11/73, p < 0.001	27/19/54, p < 0.001	30/24/46	2/0.5/98, p < 0.001	9/9/83, p < 0.001
Alcohol (units/week)	10.9 (2.4–24.5)	3 (0–14.0), p < 0.001	9.1 (2.3–23.0), p = 0.26	1.6 (0.2–6.4)	0 (0–0), p < 0.001	0.8 (0.1–3.3), p = 0.002
Green vegetables or fruit, daily/most days	1,215 (68)	953 (68), p = 0.81	272 (61), p = 0.004	409 (73.3)	223 (77.4), p = 0.19	273 (79.1), p = 0.048
Manual occupation	981 (63)	959 (77), p < 0.001	289 (85), p < 0.001	251 (53)	159 (72), p < 0.001	167 (62), p = 0.030
Years of education	10 (9–11)	12 (10–14), p < 0.001	11 (9–12), p = 0.015	10 (9–11)	11 (8–12), p = 0.97	11 (9–12), p = 0.37

Values are mean± SD, n (%), or median (25th–75th centiles). p Values for comparisons with Europeans of same sex. HbA1c indicates that baseline HbA1c is not available for African Caribbeans. CHD = coronary heart disease; DBP = diastolic blood pressure; HbA1c = glycosylated hemoglobin; HDL = high-density lipoprotein; HOMA2 = homeostasis model assessment 2; IR = insulin resistance; SBP = systolic blood pressure.

**Table 2:** Incidence Rates for First Post-Baseline CHD and Stroke Events (Nonfatal and Fatal) by Ethnicity, Sex, and Age Group at End of Follow-Up

	Person-Years of Follow-up	European		South Asian		African Caribbean	
		Men	Women	Men	Women	Men	Women
CHD							
No. of first events		473	78	526	73	67	38
Age < 55 yrs	16,631	4.3 (2.9–6.4)	1.7 (0.6–5.3)	12.6 (10.0–15.8)	3.1 (1.2–8.2)	2.1 (0.5–8.4)	1.0 (0.1–7.3)
Age 56–70 yrs	41,644	17.5 (15.6–19.8)	8.7 (6.5–11.7)	26.7 (23.9–29.8)	19.1 (14.5–25.2)	8.6 (6.1–12.1)	5.6 (3.5–9.0)
Age 70+ yrs	12,448	34.7 (29.9–40.2)	16.6 (11.6–23.8)	50.2 (42.6–59.2)	33.5 (21.1–53.2)	23.8 (16.8–33.6)	22.3 (14.4–34.6)
Stroke							
No. of first events		139	34	130	27	44	27
Age < 55 yrs	16,877	0.3 (0.1–1.4)	0	1.2 (0.6–2.4)	0.8 (0.1–5.4)	3.2 (1.0–9.9)	0
Age 56–70 yrs	44,910	3.4 (2.6–4.4)	3.1 (1.9–5.0)	5.4 (4.3–6.8)	5.1 (3.0–8.5)	3.8 (2.3–6.3)	4.9 (2.9–8.1)
Age 70+ yrs	15,006	12.6 (10.0–16.0)	9.2 (5.8–15.0)	12.9 (10.0–17.0)	16.9 (9.3–30.0)	17.5 (12.0–26.0)	12.5 (7.1–22.0)

Data are rates/1,000 person-years (95% confidence interval). Abbreviation as in Table 1.

**Table 3:** Coronary Heart Disease Events: Subhazard Ratios for First Post-Baseline Events (Nonfatal and Fatal) During 20.5 Years of Follow-Up in South Asians and African Caribbeans Compared With Europeans

	Europeans	South Asians	African Caribbeans
Unadjusted	Reference group	1.61 (1.43–1.80), p < 0.001	0.58 (0.47–0.71), p < 0.001
Adjusted for			
Age		1.77 (1.58–1.99), p < 0.001	0.59 (0.48–0.72), p < 0.001
Sex and age (model 1)		1.70 (1.52–1.91), p < 0.001	0.64 (0.52–0.79), p < 0.001
Model 1+			
Smoking (never, former, current)		1.98 (1.72–2.27), p < 0.001	0.68 (0.55–0.84), p = 0.001
Baseline CHD		1.73 (1.54–1.94), p < 0.001	0.68 (0.55–0.84), p < 0.001
Diabetes		1.55 (1.38–1.75), p < 0.001	0.60 (0.49–0.75), p < 0.001
SBP/treated hypertension⁎		1.62 (1.44–1.82), p < 0.001	0.58 (0.47–0.71), p < 0.001
Waist-to-hip ratio		1.45 (1.28–1.64), p < 0.001	0.59 (0.48–0.73), p < 0.001
Waist-to-thigh ratio		1.49 (1.32–1.69), p < 0.001	0.72 (0.58–0.88), p = 0.002
Body mass index		1.71 (1.52–1.92), p < 0.001	0.61 (0.50–0.75), p < 0.001
Total cholesterol		1.78 (1.58–2.00), p < 0.001	0.71 (0.57–0.87), p = 0.002
Triglycerides		1.60 (1.42–1.80), p < 0.001	0.71 (0.58–0.88), p = 0.002
HDL and LDL cholesterol		1.68 (1.48–1.90), p < 0.001	0.74 (0.60–0.92), p = 0.008
Fasting glucose†		1.56 (1.38–1.75), p < 0.001	0.59 (0.48–0.73), p < 0.001
HOMA2 IR†		1.51 (1.34–1.72), p < 0.001	0.58 (0.47–0.72), p < 0.001
HbA1c‡		1.48 (1.28–1.70), p < 0.001	Not available
Alcohol consumption		1.67 (1.49–1.88), p < 0.001	0.64 (0.52–0.79), p < 0.001
Green vegetable/fruit consumption		1.70 (1.51–1.91), p < 0.001	0.65 (0.53–0.80), p < 0.001
Physical activity		1.70 (1.51–1.91), p < 0.001	0.66 (0.53–0.81), p < 0.001
Years of education		1.76 (1.56–1.99), p < 0.001	0.66 (0.54–0.82), p < 0.001
Manual occupation		1.68 (1.49–1.90), p < 0.001	0.65 (0.53–0.80), p < 0.001
Multivariable			
Include covariates with largest effects on ethnic differences			
Model 1+			
Waist-to-hip ratio and HbA1c,‡ smoking		1.49 (1.25–1.77), p < 0.001	—
Waist-to-thigh ratio, LDL + HDL cholesterol, SBP/treated hypertension†, HOMA IR		—	0.74 (0.59–0.93), p = 0.011
Composite models			
Framingham variables§		1.77 (1.53–2.05), p < 0.001	0.66 (0.52–0.82), p < 0.001
INTERHEART variables∥		1.52 (1.32–1.74), p < 0.001	0.65 (0.52–0.82), p < 0.001
INTERHEART variables (subset)¶		1.52 (1.26–1.85), p < 0.001	0.68 (0.52–0.89), p = 0.004

Data are rates/1,000 person-years (95% confidence interval). Abbreviations as in Table 1.

⁎ Tertiles 1–3 (= 4 for those with treated hypertension).

† Tertiles 1–3 (= 4 for those with diabetes).

‡ HbA1c available only for 1,422 Europeans and 1,151 South Asians (age- and sex-adjusted subhazard ratio in this group, 1.68 [1.46–1.92]).

§ Framingham variables: age, sex, diabetes, current smoking, total and HDL cholesterol, SBP and treated hypertension (complete data available for multivariable adjustment 97% Europeans, 96% South Asians, 95% African Caribbeans).

∥ Age, sex, current smoking, diabetes, treated hypertension, HDL and LDL cholesterol, waist-to-hip ratio (sex-specific tertiles), alcohol consumption, daily green vegetable/fruit consumption, tertiles of physical activity (complete data available for multivariable adjustment for 93% Europeans, 90% South Asians, 89% African Caribbeans).

¶ Age, sex, current smoking, diabetes, treated hypertension, apolipoprotein B-to-A1 ratio, waist-to-hip ratio (sex-specific tertiles), alcohol consumption, daily green vegetable/fruit consumption, tertiles of physical activity. Apolipoprotein data were available only for 1,147 Europeans, 688 South Asians, and 514 African Caribbeans (age- and sex-adjusted subhazard ratios in this subsample: South Asian vs. European: 1.77 [1.49–2.10], p < 0.001, African Caribbean vs. European: 0.65 [0.51–0.84]).

**Table 4:** Stroke: Subhazard Ratios for First Post-Baseline Events (Nonfatal and Fatal) During 20.5 Years of Follow-Up in South Asians and African Caribbeans Compared With Europeans

	Europeans	South Asians	African Caribbeans
Without diabetes at baseline			
Unadjusted	Reference group	0.93 (0.71–1.20), p = 0.56	1.07 (0.76–1.52), p = 0.69
Adjusted for			
Age		1.13 (0.87–1.46), p = 0.36	1.08 (0.77–1.53), p = 0.65
Sex and age		1.12 (0.86–1.40), p = 0.38	1.12 (0.79–1.60), p = 0.52
With diabetes at baseline			
Unadjusted		1.72 (1.01–2.92), p = 0.047	2.03 (1.12–3.67), p = 0.020
Adjusted for			
Age		1.96 (1.15–3.33), p = 0.013	2.30 (1.25–4.22), p = 0.007
Age plus			
Sex		1.97 (1.16–3.35), p = 0.012	2.21 (1.14–4.30), p = 0.019
Smoking (never, former, current)		1.68 (0.94–2.98), p = 0.078	2.41 (1.22–4.78), p = 0.011
Baseline stroke		1.96 (1.15–3.33), p = 0.014	2.01 (1.09–3.70), p = 0.025
SBP/treated hypertension⁎		1.96 (1.14–3.35), p = 0.014	2.01 (1.07–3.81), p = 0.031
Waist-to-hip ratio		2.04 (1.19–3.50), p = 0.009	2.32 (1.27–4.26), p = 0.006
Waist-to-thigh ratio		1.96 (1.15–3.35), p = 0.013	2.51 (1.23–5.12), p = 0.012
Body mass index		1.82 (1.07–3.08), p = 0.027	2.26 (1.23–4.15), p = 0.009
Total cholesterol		2.11 (1.22–3.65), p = 0.008	2.60 (1.37–4.96), p = 0.004
Triglycerides		1.94 (1.13–3.31), p = 0.016	2.53 (1.37–4.67), p = 0.003
HDL and LDL cholesterol		1.92 (1.06–3.45), p = 0.030	2.13 (1.08–4.19), p = 0.029
Fasting glucose†		1.94 (1.14–3.31), p = 0.093	2.29 (1.25–4.21), p = 0.009
HOMA2 IR†		1.98 (1.11–3.56), p = 0.022	2.45 (1.30–4.61), p = 0.006
HbA1c§		1.71 (0.82–3.57), p = 0.150	Not available
Alcohol consumption		1.94 (1.12–3.34), p = 0.018	2.19 (1.13–4.24), p = 0.021
Green vegetable/fruit consumption		1.91 (1.12–3.27), p = 0.017	2.21 (1.13–4.29), p = 0.020
Physical activity		2.01 (1.18–3.43), p = 0.010	2.34 (1.26–4.34), p = 0.007
Years of education		2.12 (1.24–3.65), p = 0.006	2.25 (1.22–4.13), p = 0.009
Manual occupation		1.86 (1.09–3.19), p = 0.023	2.24 (1.19–4.19), p = 0.012
Multivariable models (include covariates with largest effects on ethnic differences)			
Smoking, years of education		1.81 (1.01–32.4), p = 0.045	—
Total cholesterol, baseline stroke, SBP/treated hypertension†		—	2.32 (1.16–4.65), p = 0.017
Composite models			
Framingham variables‡		2.05 (1.14–3.71), p = 0.017	2.04 (0.93–4.47), p = 0.074
INTERHEART variables∥		1.95 (1.05–3.63), p = 0.034	1.62 (0.71–3.65), p = 0.25

Data are rates/1,000 person-years (95% confidence interval). Abbreviations as in Table 1.

⁎ Tertiles 1–3 (= 4 for those with treated hypertension).

† Tertiles 1–3 (= 4 for those with diabetes).

§ HbA1c available only for 72 Europeans and 227 South Asians; age-adjusted subhazard ratio in this group with diabetes: 1.70 (0.82–3.51).

‡ Age, sex, (diabetes), current smoking, total and HDL cholesterol, SBP and treated hypertension (complete data available for multivariable adjustment for 97% Europeans, 96% South Asians, and 95% African Caribbeans).

∥ Age, sex, current smoking, (diabetes), treated hypertension, HDL and LDL cholesterol, waist-to-hip ratio (sex-specific tertiles), alcohol consumption, daily green vegetables/fruit, tertiles of physical activity (complete data available for multivariable adjustment for 93% Europeans, 90% South Asians, and 89% African Caribbeans).
