# Supplementary material for: The Relationship Between Metabolic Risk Factors and Incident Cardiovascular Disease in Europeans, South Asians, and African Caribbeans: SABRE (Southall and Brent Revisited)—A Prospective Population-Based Study
Source: J Am Coll Cardiol. 2013 Apr 30;61(17):1777–86. doi: 10.1016/j.jacc.2012.12.046 (PMC3677086; doi:10.1016/j.jacc.2012.12.046)
Supplement: Online Appendix, Online Tables 1 and 2 [file mmc1.docx]

Online Table 1. Baseline characteristics of those lost to follow-up ( p values compare those with and without follow-up within ethnic group)

|  | European | Indian Asian | African Caribbean |
| --- | --- | --- | --- |
| N(%) | 297 (13%) | 193 (11%) | 171(21%) |
| Age | 51.9±7.1, p=0.008 | 51.4±7.5, p=0.42 | 53.8±5.6, p=0.39 |
| Male Sex | 223(75%), p=0.64 | 160(83%), p=0.98 | 106(62%), p=0.11 |
| Diabetes | 16(5%), p=0.47 | 33(17%), p=0.18 | 27(16%), p=0.25 |
| Treated hypertension | 15(5%), p=0.013 | 25(13%), p=0.87 | 39(23%), p=0.84 |
| SBP, mm Hg | 121±16, p=0.32 | 125±17, p=0.92 | 129±17, p=0.24 |
| Fasting glucose | 5.46(5.35, 5.58),p=0.24 | 5.91(5.68, 6.14),p=0.77 | 5.84(5.62, 6.07),p=0.16 |
| Triglycerides | 1.76(1.73, 1.79), p=0.20 | 1.73(1.59, 1.89), p=0.63 | 1.08(1.01, 1.16),p=0.39 |
| HDL cholesterol | 1.32(1.28, 1.37), p=0.98 | 1.16(1.11, 1.21), p=0.19 | 1.56(1.49, 1.62),p=0.089 |
| Waist circumference | 89.3±12.2, p=0.13 | 91.0±11.4, p=0.16 | 89.2±10.3, p=0.90 |
| Waist: hip ratio | 0.91±0.09, p=0.71 | 0.96±0.09, p=0.51 | 0.91±0.08, p=0.33 |
| Pre-baseline CHD | 10(3%), p=0.038 | 10(5%), p=0.70 | 2(1%), p=0.18 |
| Pre-baseline stroke | 1(0.3%), p=0.12 | 6(3%), p=0.21 | 2(1%), p=0.21 |
|  |  |  |  |

Online Table 2. Age adjusted competing risks regression output for baseline diabetes as a predictor of stroke events, plus ethnicity- diabetes interaction (Includes all fatal and non-fatal stroke events whether directly or indirectly identified. Competing risk = death from any other cause)

|  | Subhazard ratio | Standard error | Z | P | 95% confidence interval |
| --- | --- | --- | --- | --- | --- |
| Subhazard ratio for baseline diabetes within ethnic group |  |  |  |  |  |
| Europeans | 1.27 | 0.33 | 0.94 | 0.35 | 0.77, 2.12 |
| Indian Asians | 2.50 | 0.41 | 5.56 | <0.001 | 1.81, 3.45 |
| African Caribbeans | 2.97 | 0.73 | 4.40 | <0.001 | 1.83, 4.82 |
|  |  |  |  |  |  |
|  |  |  |  |  |  |
| Ethnicity–diabetes interaction†:  Indian Asians  African Caribbeans | 1.88  2.29 | 0.57  0.81 | 2.07  2.34 | 0.038  0.019 | 1.04, 3.41  1.15, 4.59 |

†Europeans=reference group
